# Gypenosides Attenuates CORT-Induced Ferroptosis via Inhibiting TNF-α/NF-κB Signaling Pathway in PC12 Cells

**DOI:** 10.3390/molecules30102103

**Published:** 2025-05-09

**Authors:** Lingling Dai, Jinghui Peng, Manyu Zhang, Yulin Hu, Zhicheng Gao, Jibin Wang, Haiyang Zhang, Shoujun Li

**Affiliations:** College of Veterinary Medicine, South China Agricultural University, Guangzhou 510642, China

**Keywords:** gypenosides, ferroptosis, chronic stress, lipid peroxidation, iron homeostasis

## Abstract

Chronic stress can lead to nervous system dysfunction and depression-like behaviors in animals. Gypenosides can improve chronic stress-induced neuronal damage, but the protective mechanism remains poorly understood. This study aims to investigate the effect and mechanism of gypenosides on chronic stress-induced neuronal ferroptosis. Therefore, we established a chronic stress-induced neuronal damage model in vitro using corticosterone to induce PC12 cell injury. We demonstrated that ferroptosis inhibitors DFO and Ferrostatin-1 alleviated corticosterone-induced cell death in PC12 cells by reducing iron accumulation, lipid peroxidation, and increasing cell viability. Meanwhile, gypenosides attenuated ferroptosis agonist Erastin-induced ferroptosis in PC12 cells. Then, gypenosides ameliorated corticosterone-induced ferroptosis in PC12 cells. In terms of molecular mechanisms, gypenosides decreased the expression of Hepcidin and DMT1, and increased the expression of Ferritin and FPN1, thereby improving corticosterone-induced iron homeostasis disorders and iron accumulation. Moreover, gypenosides improved corticosterone-induced lipid peroxidation by inhibiting GLS2 expression, upregulating the expression of SLC7A11 and glutathione peroxidase 4, and reducing glutamate accumulation and GSH depletion. Gypenosides also reduced corticosterone-induced release of inflammatory cytokines, the expression of TNFR1, and the phosphorylation of NF-κB and p53 in PC12 cells. These findings indicate that gypenosides attenuate corticosterone-induced ferroptosis by inhibiting TNF-α/NF-κB signaling pathway in PC12 cells.

## 1. Introduction

In veterinary clinical practice, chronic stress can cause nervous system dysfunction, resulting in multiple system dysfunction and abnormal behaviors in chickens, dogs, pigs, and other animals. Studies have shown that chronic stress can lead to abnormal behaviors, such as non-feeding, chewing, and tail biting in pigs [1,2]. Subsequently, chronic stress reduces feed intake, feed conversion rate, performance, and product quality of animals, resulting in varying degrees of economic losses to animal husbandry. Studies demonstrate that prolonged stress triggers excessive activation of the hypothalamic-pituitary-adrenal (HPA) axis, which can lead to abnormal increases in glucocorticoids, resulting in nerve cell damage and depression-like behaviors in animals [3,4]. Chronic stress can induce abnormal activation of the HPA axis, increased corticosterone (CORT) levels, and decreased hippocampal neuron density, leading to depression-like behaviors in pigs [1]. However, the pathogenesis and molecular mechanisms of chronic stress-induced neuronal damage need to be further elucidated.

Ferroptosis is a newly defined type of programmed cell death and is characterized by the accumulation of massive iron-dependent lipid peroxides (LPO) and reactive oxygen species (ROS) [5,6]. The specific inhibitors, such as Ferrostatin-1 and iron chelator deferoxamine (DFO), can inhibit ferroptosis [5]. Our previous study showed that Ferrostatin-1 and DFO inhibited cell death by alleviating the iron and LPO accumulation in microglia [7]. Existing studies have shown that a variety of biological processes are closely linked to ferroptosis, including inflammatory response, iron homeostasis imbalance, glutathione (GSH) depletion, and Glutathione Peroxidase 4 (GPX4) inactivation, which lead to the accumulation of LPO and ROS, and cell death [8,9]. Iron homeostasis is essential for the survival of nerve cells [10]. Previous studies have shown that chronic stress can lead to neuroinflammation, which regulates iron metabolism-related proteins and causes iron accumulation in nerve cells [11,12]. The imbalance of iron homeostasis leads to the excessive accumulation of LPO, which is a prominent feature of ferroptosis [9]. In addition, chronic stress can cause abnormal glutamate metabolism in the brain, resulting in excitatory neurotoxicity and further damage to neurons [13,14]. Chronic stress can also increase serum CORT and nitric oxide contents and reduce the expression of neurotransmitters, which can cause abnormal behavior in pigs [15]. Our previous study demonstrates that chronic stress exacerbates nano-aluminum particles-induced hippocampal neurons ferroptosis in rats [11]. Therefore, inhibiting neuronal ferroptosis might be a therapeutic approach for mitigating chronic stress-induced neuronal damage. However, further investigation is required to identify the therapeutic agents and elucidate the specific protective mechanisms.

Gypenosides (GPs), a group of tetracyclic or pentacyclic triterpenoid saponins isolated from *Gynostemma pentaphyllum*, share structural similarities with certain ginsenosides but exhibit a distinct bioactive profile [16]. Gypenosides have been shown to exhibit antioxidant, neuroprotective, anti-inflammatory, and anti-depression properties [17,18]. It can not only be used as plant feed additives for livestock and poultry to promote animal growth and improve the quality of livestock products but also can be used as a pharmaceutical formula to participate in the prevention and control of livestock and poultry diseases. The existing study has shown that GPs can significantly modulate inflammatory cytokines secretion, increase GSH content, and reduce the production of LPO and ROS, thereby alleviating oxidative damage in rats with chronic cerebral ischemia [19]. Recent studies have shown that gypenoside A ameliorates high-glucose-induced retinal microvasculopathy by inhibiting ferroptosis [16]. Nevertheless, the protective effects and mechanisms of GPs on neuronal ferroptosis induced by chronic stress remain to be elucidated.

Inflammatory cytokines interleukin (IL)-6 and tumor necrosis factor (TNF)-α can disturb the metabolism of iron by activating nuclear factor kappa B (NF-κB) [9]. TNF-α and NF-κB are potential therapeutic targets for neuronal damage [20,21]. The TNF-α/NF-κB signaling pathway plays multiple key roles in the regulation of neuronal damage caused by programmed cell death [21,22]. The activation of TNF-α/NF-κB signaling pathway can increase Hepcidin expression, thereby increasing DMT1 and decreasing FPN1 expression [23]. Moreover, NF-κB activation is essential for neuronal ferroptosis [24]. TNF-α-induced NF-κB can activate the tumor suppressor p53, which is crucial in the regulation of ferroptosis [9]. Moreover, p53 can interfere with the metabolism of glutamate (Glu) and cause GSH depletion, which eventually leads to neurons’ ferroptosis [25]. Previous studies showed that GPs can inhibit the activation of NF-κB and reduce the production of inflammatory cytokines IL-6, IL-1β, and TNF-α, thereby alleviating chronic stress-induced hippocampal neuroinflammation and depression-like behavior in rats [18]. However, it is currently unknown whether GPs inhibit chronic stress-induced neurons’ ferroptosis by TNF-α/NF-κB signaling pathway.

Overactivation of the HPA axis is a key marker of the chronic stress response, and CORT is widely used to establish in vitro chronic stress models [26]. PC12 cells, derived from rat pheochromocytoma, are extensively utilized in neuroscience research, including studies on neurotoxicity, neuroinflammation, and neuroprotection [27]. Therefore, we used CORT to induce PC12 cell injury to establish a chronic stress-induced neuronal injury model in vitro. Specific ferroptosis inhibitors, Ferrostatin-1 and DFO, were used to determine whether CORT induced ferroptosis in PC12 cells. After that, a specific ferroptosis agonist, Erastin, was used to determine the protective effect of GPs on ferroptosis in PC12 cells. Finally, PC12 cells were pretreated with GPs to explore whether GPs could improve CORT-induced ferroptosis in PC12 cells by inhibiting TNF-α/NF-κB signaling pathway. This study provides a theoretical and experimental basis for the use of GPs to prevent and treat animal stress. At the same time, it provides drug targets for screening anti-stress drugs in livestock farms.

## 2. Results

### 2.1. Effects of DFO and Ferrostatin-1 on CORT-Induced PC12 Cells Viability

**Figure 1:**
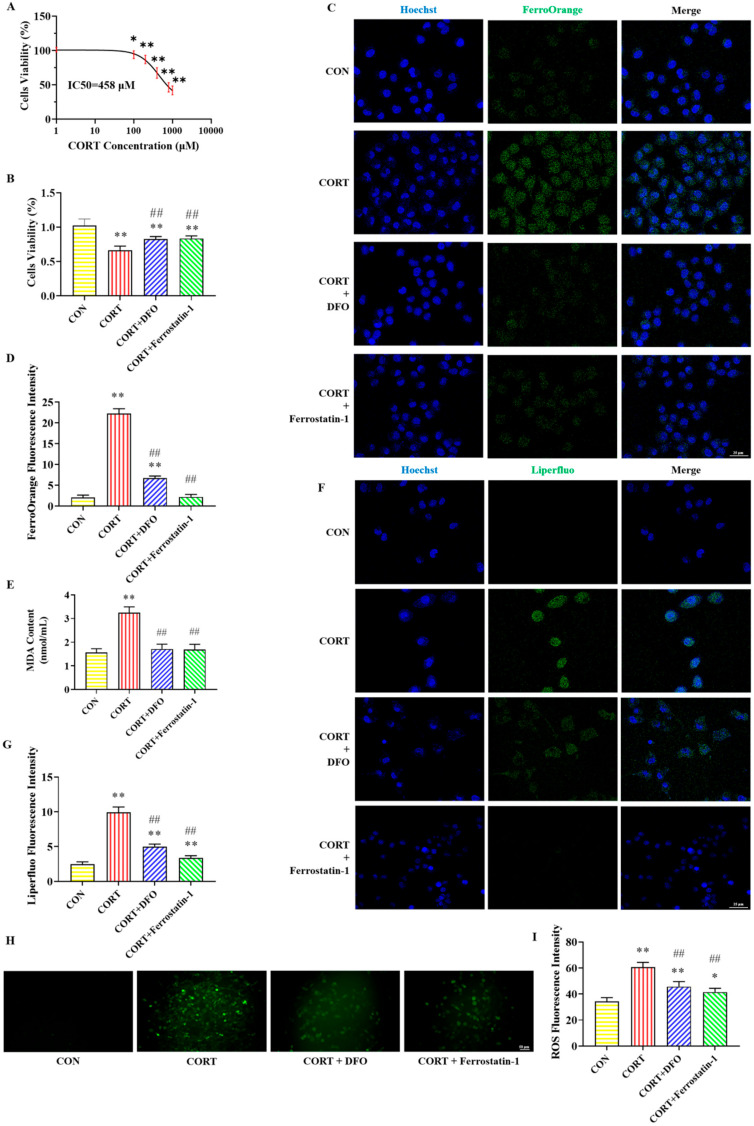
Effects of DFO and Ferrostatin-1 on CORT-induced ferroptosis in PC12 cells. (**A**) PC12 cells’ viability after treatment with different concentrations of CORT. (**B**) The viability of PC12 cells after different treatments. (**C**) Representative FerroOrange (green) positive cells immunofluorescence images at 400× magnification. Nuclei were stained with Hoechst (blue) in PC12 cells. Scale bar = 25 μm. (**D**) The fluorescence intensity of iron. (**E**) The content of MDA. (**F**) Representative Liperfluo (green) positive cells immunofluorescence images at 400× magnification. Nuclei were stained with Hoechst (blue) in PC12 cells. Scale bar = 25 μm. (**G**) The fluorescence intensity of LPO. (**H**) Representative ROS immunofluorescence images of PC12 cells at 200× magnification. Scale bar = 50 μm. (**I**) The fluorescence intensity of ROS. All data are presented as the mean ± SD (n = 6). * *p* < 0.05 and ** *p* < 0.01 versus CON group. ^##^ *p* < 0.01 versus CORT group.

The cytotoxicity of various CORT concentrations on PC12 cells is presented in Figure 1A. The IC_50_ for CORT in PC12 cells was determined to be 458 μM using GraphPad Prism 10.1.2 software. We consulted the literature on the concentration range of CORT and found that the range selected for establishing the CORT-induced PC12 cell injury model was mainly between 100 μM and 800 μM. Consequently, 400 μM CORT (approximately IC_50_) was chosen for the following experiments. With this treatment, the survival rate of PC12 cells significantly decreased.

The effects of various drug treatments on the viability of PC12 cells are illustrated in Figure 1B. In contrast to the CON group, the cells’ viability was decreased in the CORT group. Compared with the CORT group, the cells’ viability significantly increased in the CORT + DFO and CORT + Ferrostatin-1 groups. The results show that DFO and Ferrostatin-1 can improve the survival rate of CORT-induced PC12 cells.

### 2.2. Effect of DFO and Ferrostatin-1 on CORT-Induced PC12 Cells Iron Accumulation

The immunofluorescent staining results of iron in PC12 cells are shown in Figure 1C,D. Compared with the CON group, the fluorescence intensity of FerroOrange (green) in PC12 cells was obviously increased in the CORT group, while the fluorescence intensity of FerroOrange was significantly decreased in the CORT + DFO and CORT + Ferrostatin-1 groups compared to the CORT group. These findings suggest that DFO and Ferrostatin-1 can improve CORT-induced PC12 cells’ iron accumulation.

### 2.3. Effect of DFO and Ferrostatin-1 on CORT-Induced Lipid Peroxidation in PC12 Cells

Figure 1E–I illustrate the detection results of lipid peroxidation-related indicators in PC12 cells. Compared with the CON group, the MDA content and the fluorescence intensity of Liperfluo and ROS were obviously elevated in the CORT group. In contrast, these lipid peroxidases were significantly lower in the CORT + DFO and CORT + Ferrostatin-1 groups than in the CORT group. These results suggest that CORT can cause lipid peroxidation, while DFO and Ferrostatin-1 alleviate lipid peroxide accumulation in PC12 cells.

### 2.4. Effect of GPs on Erastin-Induced Viability of PC12 Cells

**Figure 2:**
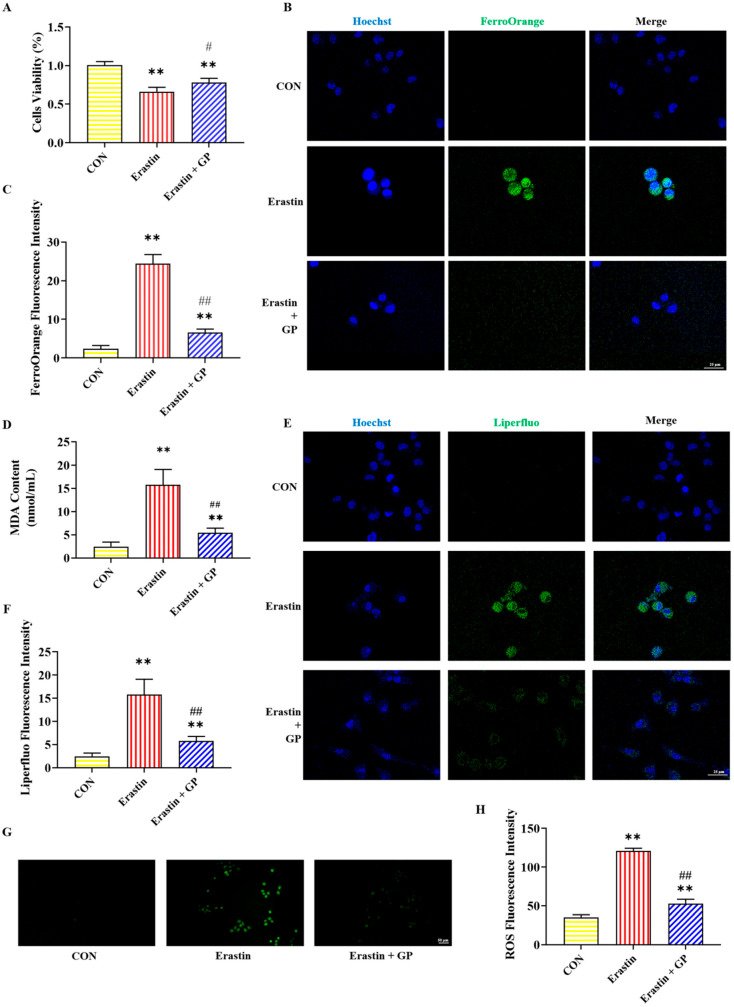
Effects of GPs on Erastin-induced ferroptosis in PC12 cells. (**A**) The viability of PC12 cells after different treatments. (**B**) Representative FerroOrange (green) positive cells immunofluorescence images at 400× magnification. Nuclei were stained with Hoechst (blue) in PC12 cells. Scale bar = 25 μm. (**C**) The fluorescence intensity of iron. (**D**) The content of MDA. (**E**) Representative Liperfluo (green) positive cells immunofluorescence images at 400× magnification. Nuclei were stained with Hoechst (blue) in PC12 cells. Scale bar = 25 μm. (**F**) The fluorescence intensity of LPO. (**G**) Representative ROS immunofluorescence images of PC12 cells at 200× magnification. Scale bar = 50 μm. (**H**) The fluorescence intensity of ROS. All data are presented as the mean ± SD (n = 6). ** *p* < 0.01 versus CON group. ^#^ *p* < 0.05 and ^##^ *p* < 0.01 versus CORT group.

In the Erastin group, PC12 cell viability was significantly lower than in the CON group, while it was significantly higher in the Erastin + GP group than in the Erastin group (Figure 2A). These results suggest that GPs enhance the Erastin-induced PC12 cells’ viability.

### 2.5. Effect of GPs on Erastin-Induced Iron Accumulation in PC12 Cells

The immunofluorescent staining results of iron in PC12 cells are shown in Figure 2B,C. Compared with the CON group, the FerroOrange fluorescence intensity in the Erastin group showed a significant increase in PC12 cells. Compared with the Erastin group, the FerroOrange fluorescence intensity in the Erastin + GP group was significantly decreased. These results indicate that GPs can mitigate Erastin-induced PC12 cell iron accumulation.

### 2.6. Effect of GPs on Erastin-Induced Lipid Peroxidation in PC12 Cells

Figure 2D–H illustrate the detection results of lipid peroxidation-related indicators in PC12 cells. The MDA content and the fluorescence intensity of Liperfluo and ROS were sharply heightened in the Erastin group compared with the CON group, while those were significantly reduced in the Erastin + GP group compared with the Erastin group. These results indicate that GPs can improve Erastin-induced lipid peroxidation in PC12 cells.

### 2.7. Effect of GPs on CORT-Induced PC12 Cells Viability

**Figure 3:**
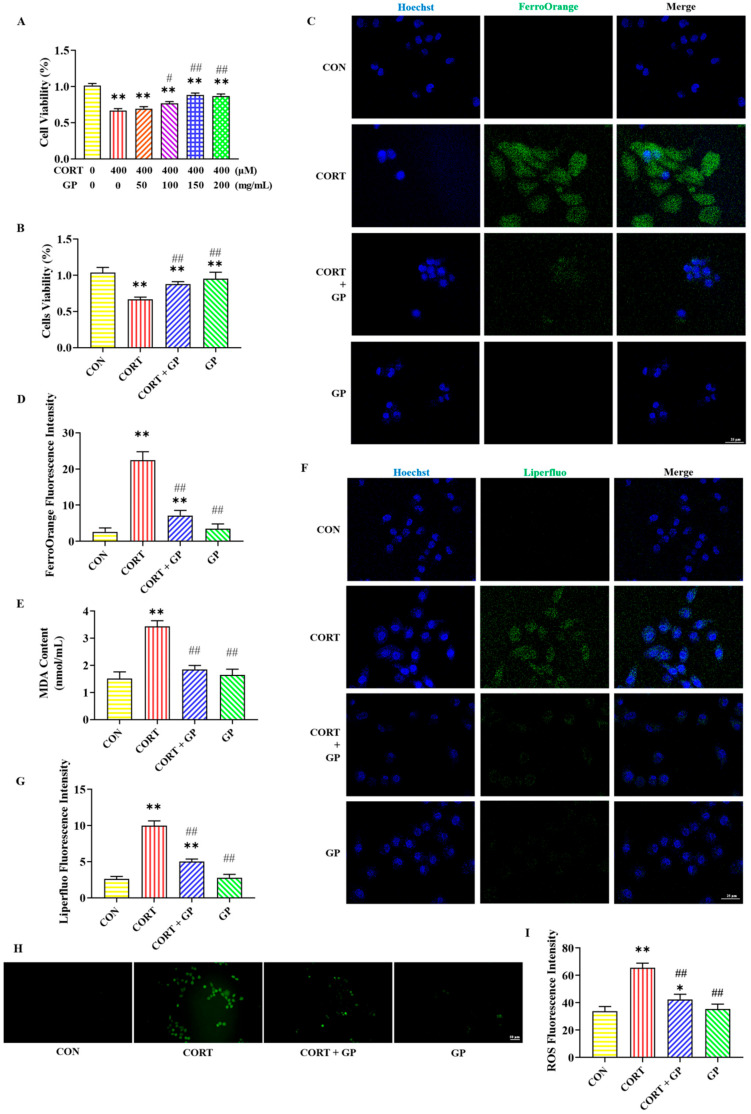
Effects of GPs on CORT-induced ferroptosis in PC12 cells. (**A**) Effects of different concentrations of GPs on the viability of PC12 cells treated with 400 μM CORT. (**B**) The viability of PC12 cells after different treatments. (**C**) Representative FerroOrange (green) positive cells immunofluorescence images at 400× magnification. Nuclei were stained with Hoechst (blue) in PC12 cells. Scale bar = 25 μm. (**D**) The fluorescence intensity of iron. (**E**) The content of MDA. (**F**) Representative Liperfluo (green) positive cells immunofluorescence images at 400× magnification. Nuclei were stained with Hoechst (blue) in PC12 cells. Scale bar = 25 μm. (**G**) The fluorescence intensity of LPO. (**H**) Representative ROS immunofluorescence images of PC12 cells at 200× magnification. Scale bar = 50 μm. (**I**) The fluorescence intensity of ROS. All data are presented as the mean ± SD (n = 6). * *p* < 0.05 and ** *p* < 0.01 versus CON group. ^#^ *p* < 0.05 and ^##^ *p* < 0.01 versus CORT group.

Figure 3A,B illustrate the effect of GPs on the viability of PC12 cells treated with CORT. Treatment with 400 μM CORT significantly reduced the survival rate of PC12 cells. However, the addition of 100, 150, and 200 mg/mL GPs significantly increased the survival rate of PC12 cells. Moreover, 150 mg/mL GPs had the best protective effect and no obvious damage to cells, and the protective effect was not significantly improved when the dose was higher than this. These results suggest that GPs enhance CORT-induced PC12 cells’ viability. Therefore, 150 mg/mL GPs was chosen for subsequent cell experiments.

### 2.8. Effect of GPs on CORT-Induced PC12 Cells Iron Accumulation

The immunofluorescent staining results of iron in PC12 cells are shown in Figure 3C,D. Compared to the CON group, the FerroOrange fluorescence level was sharply increased in the CORT group. Contrasted with the CORT group, the FerroOrange fluorescence level was obviously decreased in the CORT + GP group. These results indicate that GP can improve CORT-induced PC12 cells’ iron accumulation.

### 2.9. Effect of GPs on CORT-Induced PC12 Cells Lipid Peroxidation

Figure 3E–I present the detection results of lipid peroxidation-related indicators in PC12 cells. The MDA content and the fluorescence intensity of Liperfluo and ROS were significantly higher in the CORT group than in the CON group, while those indicators were significantly lower in the CORT + GP group than in the CORT group. These results indicate that GPs can improve CORT-induced PC12 cells lipid peroxidation.

### 2.10. Effect of GPs on CORT-Induced PC12 Cells Iron Homeostasis

**Figure 4:**
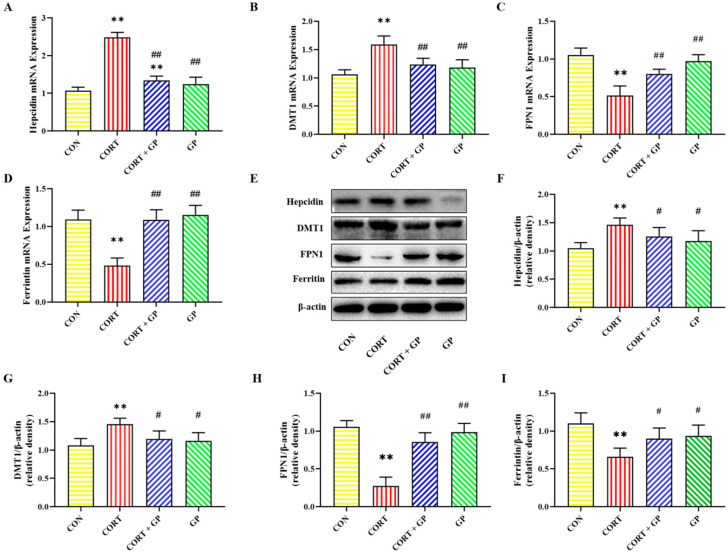
Effects of GPs on CORT-induced iron metabolism in PC12 cells. (**A**–**D**) The relative mRNA expression of *Hepcidin*, *DMT1*, *FPN1*, and *Ferritin*. (**E**) The relative protein expression of Hepcidin, DMT1, FPN1, and Ferritin. (**F**–**I**) The protein quantitative analysis of Hepcidin, DMT1, FPN1, and Ferritin. All data are presented as the mean ± SD (n = 6). ** *p* < 0.01 versus CON group. ^#^ *p* < 0.05 and ^##^ *p* < 0.01 versus CORT group.

The mRNA and protein expression levels of iron homeostasis-related proteins in PC12 cells are shown in Figure 4. Compared with the CON group, the mRNA and protein expression levels of Hepcidin and DMT1 were significantly increased, while the mRNA and protein expression levels of FPN1 and Ferritin were significantly decreased in the CORT group. Compared with the CORT group, the mRNA and protein expression levels of Hepcidin and DMT1 were significantly decreased, while the mRNA and protein expression levels of FPN1 and Ferritin were significantly increased in the CORT + GP group. These results show that GPs improve CORT-induced iron homeostasis imbalance in PC12 cells.

### 2.11. Effect of GPs on CORT-Induced PC12 Cells Glu Metabolism

**Figure 5:**
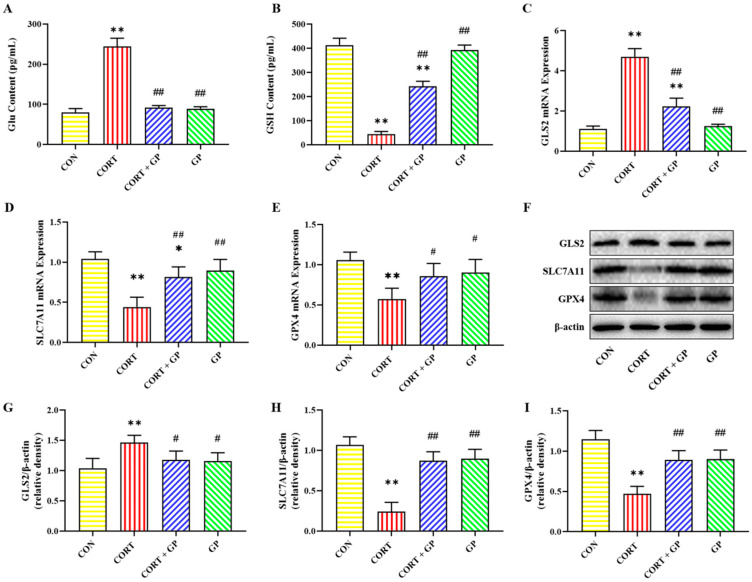
Effects of GPs on the CORT-induced Glu metabolism in PC12 cells. (**A**,**B**) The contents of Glu and GSH. (**C**–**E**) The relative mRNA expression of *GLS2*, *SLC7A11*, and *GPX4*. (**F**) The relative protein expression of GLS2, SLC7A11, and GPX4. (**G**–**I**) The protein quantitative analysis of GLS2, SLC7A11, and GPX4. All data are presented as the mean ± SD (n = 6). * *p* < 0.05 and ** *p* < 0.01 versus CON group. ^#^ *p* < 0.05 and ^##^ *p* < 0.01 versus CORT group.

Glu and GSH contents in PC12 cells are shown in Figure 5A,B. Compared with the CON group, the Glu content was significantly increased in the CORT group, while it was significantly decreased in the CORT + GP group compared with the CORT group. Compared with the CON group, the GSH content in the CORT group was significantly decreased. On the contrary, compared with the CORT group, the GSH content was significantly increased in the CORT + GP group.

The mRNA and protein expressions of Glu metabolism-related proteins in PC12 cells are shown in Figure 5C–I. Compared with the CON group, the mRNA and protein expressions of GLS2 were significantly increased, while the mRNA and protein expressions of SLC7A11 and GPX4 were significantly decreased in the CORT group. Compared with the CORT group, the mRNA and protein expressions of GLS2 were significantly decreased, while the mRNA and protein expressions of SLC7A11 and GPX4 were significantly increased in the CORT + GP group. These results indicate that GPs can significantly improve the CORT-induced Glu metabolism disorders in PC12 cells.

### 2.12. Effect of GPs on CORT-Induced Release of Inflammatory Cytokines in PC12 Cells

**Figure 6:**
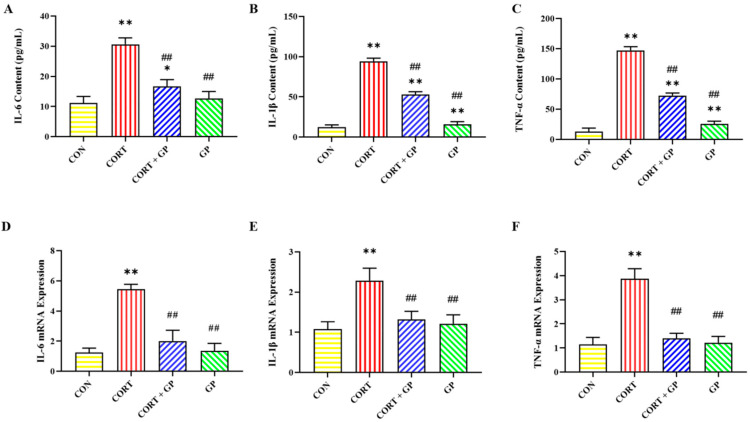
Effect of GPs on the CORT-induced release of inflammatory cytokines in PC12 cells. (**A**–**C**) The content of IL-6, IL-1β, and TNF-α in the medium supernatant. (**D**–**F**) The relative mRNA expression of *IL-6*, *IL-1β*, and *TNF-α* in PC12 cells. All data are presented as the mean ± SD (n = 6). * *p* < 0.05 and ** *p* < 0.01 versus CON group. ^##^ *p* < 0.01 versus CORT group.

The content and mRNA expression of inflammatory cytokines are shown in Figure 6. The levels and mRNA expression of *IL-6*, *IL-1β*, and *TNF-α* in the CORT group were significantly higher than those in the CON group, while these inflammatory cytokines in the CORT + GP group were significantly lower than those in the CORT group. These results suggest that GPs can inhibit the CORT-induced release of inflammatory cytokines in PC12 cells.

### 2.13. Effect of GPs on CORT-Induced TNF-α/NF-κB Signaling Pathway in PC12 Cells

**Figure 7:**
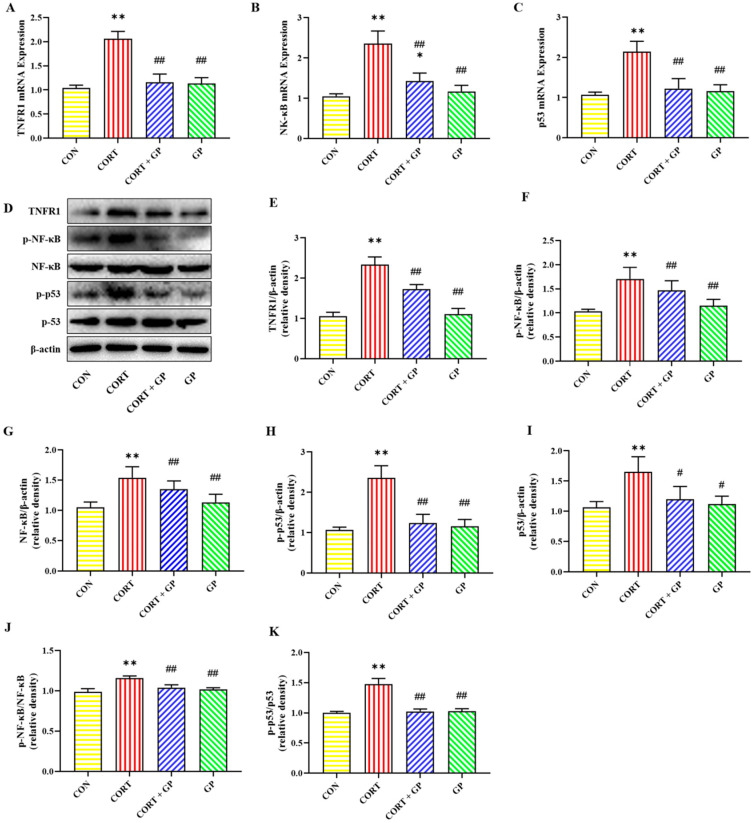
Effect of GPs on CORT-induced TNF-α/NF-κB signaling pathway in PC12 cells. (**A**–**C**) The relative mRNA expression of *TNFR1*, *NF-κB*, and *p53*. (**D**) The relative protein expression of TNFR1, p-NF-κB, NF-κB, p-p53, and p53. (**E**–**I**) The protein quantitative analysis of TNFR1, p-NF-κB, NF-κB, p-p53, and p53. (**J**) The protein expression ratio of p-NF-κB to NF-κB. (**K**) The protein expression ratio of p-p53 to p53. All data are presented as the mean ± SD (n = 6). * *p* < 0.05 and ** *p* < 0.01 versus CON group. ^#^ *p* < 0.05 and ^##^ *p* < 0.01 versus CORT group.

The mRNA expressions of *TNFR1*, *NF-κB*, and *p53* were significantly increased in the CORT group compared with the CON group, while these mRNA expressions were obviously decreased in the CORT + GP group compared with the CORT group (Figure 7A–C). Likewise, the protein expressions of TNFR1, p-NF-κB, NF-κB, p-p53, and p53 in the CORT group were significantly increased relative to those in the CON group, whereas these protein expressions were clearly declined in the CORT + GP group compared with the CORT group (Figure 7D–I). Moreover, the ratio of p-NF-κB to NF-κB and p-p53 to p53 was significantly increased in the CORT group compared with the CON group, while those ratios were significantly decreased in the CORT + GP group compared with the CORT group (Figure 7J,K). These results suggest that GPs inhibit activation of the CORT-induced TNF-α/NF-κB signaling pathway in PC12 cells.

## 3. Materials and Methods

### 3.1. Cell Culture and Drug Treatments

PC12 cells were sourced from the Henan Engineering Technology Research Center of Industrial Microbial Strains. These cells were cultured in DMEM (Dulbecco’s Modified Eagle Medium) from Gibco (Thermo, Waltham, MA, USA), with the addition of 10% fetal bovine serum (FBS, BI, Beit Haemek, Israel) and 100 U/mL penicillin-streptomycin. Incubation was carried out at 37 °C in a humidified chamber (Thermo, Waltham, MA, USA) containing 5% CO_2_. Cells were cultured until they achieved 70–80% confluency in T25 flasks. Before treatment, PC12 cells were seeded at a density of 5 × 10^3^ cells/mL into 6-well or 96-well plates and allowed to settle for 24 h.

Cells were classified into eight distinct groups based on various treatments: Control (CON), CORT, CORT + DFO, CORT + Ferrostatin-1, Erastin, Erastin + GP, CORT + GP, and GP groups. Cells in the CON group were cultured in the complete medium without treatment. Cells in the CORT group were incubated with 400 μM CORT (≥98%, Yuanye Bio-Technology Co., Ltd., Shanghai, China) in complete medium for 24 h. Cells in the CORT + DFO and CORT + Ferrostatin-1 groups were treated with 100 μM DFO (Sigma-Aldrich, St. Louis, MO, USA) or 10 μM Ferrostatin-1 (Sigma-Aldrich, St. Louis, MO, USA) in complete medium for 30 min, followed by 24-h exposure to 400 μM CORT. Cells in the Erastin group were treated with 20 μM Erastin (Selleck. cn, Shanghai, China) in complete medium for 24 h. In the Erastin + GP group, cells were pre-incubated with 150 μg/mL GPs (≥98%, DeSiTe Bio-Technology Co., Ltd., Chengdu, China) for 30 min and then co-treated with 20 μM Erastin for 24 h. In the CORT + GP group, cells were pre-incubated with 150 μg/mL GPs for 30 min and then co-incubated with 400 μM CORT for 24 h. Cells in the GP group were incubated with 150 μg/mL GPs in complete medium for 24 h. The concentrations of Erastin [28], Ferrostatin-1 [29], and DFO [30] were determined based on previous studies. CORT and GPs were dissolved in DMSO with final concentrations not exceeding 0.1%.

### 3.2. Cell Viability Assay

PC12 cells were distributed into 96-well plates at a density of 5 × 10^3^ cells per well in 100 μL of culture medium. After 24 h, the cells were subjected to various concentrations of CORT (0, 100, 200, 400, 800, and 1000 μM) and GPs (0, 50, 100, 150, and 200 μg/mL) for 24 h. Cell viability was assessed using the cell counting kit-8 assay (Beyotime, Shanghai, China) as according to the manufacturer’s guidelines.

### 3.3. ELISA Kits Assay

The concentrations of IL-6, IL-1β, and TNF-α in the culture supernatants of PC12 cells were measured using specific ELISA kits (Boster Biological Technology Co., Ltd., Wuhan, China) according to the manufacturer’s protocols. The levels of ROS fluorescence intensity, MDA content, and Glu and GSH concentrations in PC12 cells were measured utilizing specific assay kits (Nanjing Jiancheng Bioengineering Institute, Nanjing, China) according to the manufacturer’s instructions.

### 3.4. Iron Accumulation Assay

The FerroOrange was used to detect the accumulation of bivalent iron in PC12 cells. Detailed operation according to the manufacturer’s instructions (Dojido, Kumamoto, Japan). Fluorescence images of FerroOrange/Hoechst co-stained cells were acquired using a confocal fluorescence microscope (Leica, Wetzlar, Germany) at 400× magnification. Six non-repetitive areas in each group were photographed for statistics. Fluorescent intensity was analyzed using Image J 1.45 software.

### 3.5. LPO Fluorescence Intensity Assa0079

Liperfluo was utilized to measure the LPO fluorescence intensity in PC12 cells. Detailed operation according to manufacturer instructions (Dojido, Kumamoto, Japan). Fluorescence images of Liperfluo/Hoechst co-stained cells were acquired using a fluorescence microscope (Leica, Wetzlar, Germany) at 400× magnification. Six non-repetitive areas in each group were photographed for statistics. Fluorescent intensity was analyzed using Image J 1.45 software.

### 3.6. Western Blot Analysis 

Protein extraction and Western blot analysis were conducted as outlined in earlier studies [11,31]. The antibodies as followed: anti-Hepcidin, anti-DMT1, anti-FPN1, anti-Ferritin, anti-GLS2, anti-SLC7A11, anti-GPX4, anti-TNFR1, anti-NF-κB, anti-p-NF-κB, anti-p53, anti-p-p53 (Abcam, Cambridge, UK), and anti-β-actin (ZSGE-BIO, Beijing, China).

### 3.7. Quantitative Real-Time PCR Analysis

Total RNA was extracted from PC12 cells and converted into complementary DNA using the GoScript Reverse Transcription Kit (Promega Corporation, Madison, WI, USA). Quantitative real-time PCR was conducted on a Roche 480 Real-Time PCR System (Roche, CH) with IQ SYBR Green Supermix (Bio-Rad, San Diego, CA, USA). Glyceraldehyde 3-phosphate dehydrogenase (GAPDH) served as the internal control. Primer sequences are listed in Table 1. Specific procedures were described in our previous study [11,20].

### 3.8. Statistical Analysis

Data were expressed as mean ± standard deviation (SD) and analyzed using SPSS software (version 22.0; SPSS, Chicago, IL, USA). The Student’s *t*-test was used for two-group comparisons, while ANOVA, followed by Tukey’s *post hoc* test, was applied for multiple-group comparisons. Each experiment included six biological replicates with three technical replicates per biological replicate. Significance was considered at *p* < 0.05.

## 4. Discussion

Chronic stress can cause nerve damage in animals, resulting in neuroimmune disorders and behavioral abnormalities [1,15]. Our previous studies showed that inhibition of ferroptosis ameliorated chronic stress-induced hippocampal damage and depression-like behaviors [7,11]. However, inexpensive and effective additive drugs for the treatment of chronic stress-related neurological diseases in animal husbandry still need to be further screened. In the present study, we confirmed that GPs could inhibit chronic stress-induced neural cells’ ferroptosis in vitro by using specific ferroptosis inhibitors and agonists. Furthermore, GPs alleviated CORT-induced neural cells ferroptosis by inhibiting the TNF-α/NF-κB signaling pathway.

GPs have strong anti-inflammatory and antioxidant functions. Regarding the selection of GPs concentration and duration of treatment, we consulted the literature and found that 25–800 μg/mL of GPs could effectively alleviate hydrogen peroxide-induced rat retinal ganglion cell damage [16]. In addition, 200 μg/mL GP can inhibit Glu-induced primary cortical cell damage [32]. In this study, we found that 150 mg/mL GPs had the best protective effect and no obvious damage to cells.

Ferroptosis is a new type of programmed cell death characterized by the accumulation of massive lipid peroxides and ROS, accompanied by significant intracellular iron accumulation [5]. As ferroptosis is driven by excessive LPO, LPO is an important biomarker of ferroptosis [5]. MDA is one of the products of cell membrane lipid peroxidation, which can indirectly reflect the degree of cellular lipid peroxidation. FerroOrange and Liperfluo are divalent iron ion and LPO fluorescent probes, respectively, which are widely used for the detection of ferroptosis [33]. To further explore whether the neuronal damage induced by CORT is linked to ferroptosis, CORT-induced PC12 cells were pretreated with DFO and Ferrostatin-1 in this experiment. We found that CORT-induced iron accumulation and lipid peroxidation occurred in PC12 cells. In contrast, the DFO and Ferrostatin-1 reversed iron accumulation and lipid peroxidation. These results suggest that CORT can induce ferroptosis in PC12 cells.

Erastin is widely used as an agonist of ferroptosis [6]. Moreover, GPs have a neuroprotective effect and improve cognitive dysfunction. To determine whether the neuroprotective effect of GPs was related to ferroptosis, Erastin-induced PC12 cells were pretreated with GPs in this study. We verified that GPs could alleviate PC12 cell ferroptosis by inhibiting Erastin-induced oxidative stress and lipid peroxidation, cellular iron accumulation, and cell death.

To investigate the protective effect of GPs on CORT-induced ferroptosis in PC12 cells, we pretreated CORT-induced PC12 cells with GPs. The results showed GPs reversed the CORT-induced ferroptosis in PC12 cells. Similarly, GPs can relieve neuronal damage caused by hydrogen peroxide [16] and chronic unpredictable mild stress [18]. The above results prove that the neuroprotection of GPs is closely linked to the inhibition of ferroptosis.

Neuronal iron homeostasis is tightly regulated by ferroproteins, such as Hepcidin, DMT1, FPN1, and Ferritin [9]. Hepcidin, an important downstream effector molecule affecting iron transport between different iron pools, can negatively regulate the expression of DMT1 and FPN1 in response to stimuli such as injury, infection, and inflammation, thereby acting as a regulator of iron homeostasis [12]. DMT1 handles iron uptake, while FPN1 serves as the sole iron exit channel [9]. Additionally, Ferritin level is inversely related to intracellular iron level. Ferritin degradation leads to increased intracellular iron and aggravates neuronal damage [9]. Studies have found that chronic mild stress can increase the expression of Hepcidin, thereby increasing the expression of DMT1 and reducing the expression of FPN1 and ferritin, resulting in iron accumulation in the hippocampus [12]. Moreover, CORT induces dysregulation of iron metabolism in hippocampal neurons in vitro [26]. In this study, GPs can alleviate the CORT-induced imbalance of iron homeostasis by improving the expression of iron metabolism key proteins in PC12 cells.

Moreover, Glu metabolism is another regulatory pathway for ferroptosis [9]. Glu is formed from the breakdown of glutamine by the glutaminase and can cause a large accumulation of Glu under pathological conditions [5]. Typically, the System Xc^-^ (cystine/Glu transporter) exchanges cystine and Glu in a 1:1 ratio, and SLC7A11 is a light-chain subunit of System Xc^-^ [5]. GSH is mainly synthesized from cystine, and GSH is an important intracellular antioxidant. However, high levels of Glu inhibit the function of the System Xc^-^, thereby limiting GSH synthesis. Inhibition of GSH synthesis can cause antioxidant dysfunction and large amounts of LPO and lipid ROS accumulate, ultimately leading to neuron ferroptosis [5]. In addition, GPX4 is a negative regulator of lipid peroxidation, and GPX4 can convert potentially toxic lipid hydroperoxides to non-toxic lipid alcohols by consuming GSH. The blockage of GSH synthesis inactivates GPX4, leading to a large accumulation of LPO, finally resulting in neurons’ ferroptosis [8]. Therefore, GSH depletion and GPX4 inactivation are also the signature characteristics of ferroptosis. In this study, GPs can alleviate CORT-induced neuronal ferroptosis by improving Glu metabolism in PC12 cells.

We determined the release of inflammatory cytokines in this study to further examine the protective mechanism of GPs on CORT-induced neurons’ ferroptosis. Our results showed that GPs reduced the release and expression of inflammatory cytokines in PC12 cells. Therefore, the protective mechanism of GPs on ferroptosis is closely related to inhibiting inflammatory cytokines and corresponding signaling pathways. There is growing evidence that TNF-α/NF-κB signaling pathway can directly regulate the expression of several key genes and proteins involved in ferroptosis, such as p53 [20]. The p53 is a key regulatory factor of ferroptosis [24]. The p53 can not only up-regulate GLS2 gene expression to promote Glu synthesis but also directly inhibit the expression of SLC7A11, resulting in GSH depletion and finally leading to lipid peroxidation [25]. Our results suggested that GPs inhibited activation of the CORT-induced TNF-α/NF-κB signaling pathway in PC12 cells. Moreover, GPs decreased the phosphorylation of NF-κB and p53. Similarly, GPs play a neuroprotective role by inhibiting the release of inflammatory cytokines and the NF-κB signaling in the hippocampus [18]. Based on these findings, the inhibition of TNF-α/NF-κB signaling pathway is the key molecular mechanism by which GPs relieve CORT-induced ferroptosis in PC12 cells.

However, this study also has some limitations. The main components of GPs include Gypenoside L, Gypenoside LI, Gypenoside XLIX, and so on. The GPs used in this study were analyzed by high-performance liquid chromatography, and Gypenoside XLIX was found to be the main component, in addition to Rutin, Isorhamnetin, Quercetin, Allose, Linoleic acid, etc. All the components above are components of Gypenoside extract, but the effects of Gypenoside XLIX monomer need to be further studied. In addition, the neuroprotective mechanism of GPs may involve multiple signaling pathways in different pathological models [34]. At present, there is a lack of corresponding pathway verification and animal regression experiments. Our research group is conducting the next experiment to improve it. In the future, consideration should be given to linking research findings to the development of potential drugs.

## 5. Conclusions

This study revealed a novel neuroprotective mechanism by which GPs alleviate chronic stress-induced neuronal ferroptosis. Specifically, GPs alleviated CORT-induced ferroptosis in PC12 cells by inhibiting the TNF-α/NF-κB signaling pathway, reducing the release of inflammatory factors, alleviating the metabolism disorder and accumulation of iron, and inhibiting the phosphorylation of p53 to improve the disorder of glutamate metabolism and reduce the accumulation of lipid peroxide. This study provides a new mechanistic understanding of nerve cell death caused by chronic stress and provides experimental and theoretical bases for the use of GPs in the prevention and treatment of chronic stress in animal husbandry.

## Figures and Tables

**Table 1:** Primer sequence and amplification length of the destination fragment.

Gene	Number	Upstream and Downstream Primer Sequence	Product Length (bp)
*Hepcidin*	NM_053469.2	F: CTATCTCCGGCAACAGACGA R: TGTCTCGCTTCCTTCGCTTC	110
*DMT1*	NM_013173.2	F: TGGTTAGCGTGGCTTATCTGG R: AGTATTGCCACCGCTGGTATC	143
*FPN1*	NM_133315.2	F: TGGGAGCATCAGCAATAAC R: CAGACCAGTCCGAACAAGG	86
*Ferritin*	NM_022500.5	F: GGAACTTCACAAACTGGCTAC R: TGGATTTCACCTGCTCATT	89
*GLS2*	NM_001270786.1	F: GGGTGTCCGGTACTACTTCG R: GTTCGAGGCATCATGGTCCG	94
*GPX4*	NM_001039849.3	F: GACCTTCCCCAGACCAGCAAC R: CGCAACCCCTGTACTTATCCAG	145
*SLC7A11*	NM_001107673.3	F: TCAAATCCTTGGCCATCTGC R: ACCAATTCCTTTAGCCCATCATC	92
*IL-6*	NM_012589.2	F: CTTCTTGGGACTGATGTTG R: TACTGGTCTGTTGTGGGTG	97
*IL-1β*	NM_031512.2	F: CTCGTGGGATGATGACGACC R: AGCTTTCAGCTCACATGGGT	118
*TNF-α*	NM_012675.3	F: GCCACCACGCTCTTCTGTC R: GCTACGGGCTTGTCACTCG	149
*TNFR1*	NM_013091.2	F: CCAAGTGCCACAAAGGAACC R: GTGCCTTTATCACACACCTCG	85
*NF-κB*	NM_199267.2	F: ACTGCCGGGATGGCTTCTAT R: CTTGCTCCAGGTCTCGCTTC	105
*p53*	NM_030989.3	F: AGCGACTACAGTTAGGGGGT R: ACAGTTATCCAGTCTTCAGGGG	89
*GAPDH*	NM_017008.4	F: GGCAAGTTCAACGGCACAG R: CGCCAGTAGACTCCACGAC	142

## Data Availability

The original contributions presented in this study are included in the article. Further inquiries can be directed to the corresponding authors.
